# Supplementary material for: Associations of Exercise Habits in Adolescence and Old Age with Risk of Osteoporosis in Older Adults: The Bunkyo Health Study
Source: J Clin Med. 2021 Dec 19;10(24):5968. doi: 10.3390/jcm10245968 (PMC8707562; doi:10.3390/jcm10245968)
Supplement: Supplementary file 1 [file jcm-10-05968-s001.zip › jcm-1463184-supplementary.pdf]

Supplementary Table S1. The cumulate number of people for each sport in adolescence exercise habits, called "Bukatsudo."

	All (n=857)		Men (n=428)		Women (n=429)	
	n	(%)	n	(%)	n	(%)
Volleyball	185	(21.6)	37	(8.6)	148	(34.5)
Baseball and Softball	137	(16.0)	99	(23.1)	38	(8.9)
Tennis	132	(15.4)	45	(10.5)	87	(20.3)
Table tennis	118	(13.8)	46	(10.7)	72	(16.8)
Basketball	118	(13.8)	65	(15.2)	53	(12.4)
Track & Field	71	(8.3)	41	(9.6)	30	(7.0)
Judo	49	(5.7)	49	(11.4)	0	(0.0)
Swimming	45	(5.3)	29	(6.8)	16	(3.7)
Gymnastics	43	(5.0)	11	(2.6)	32	(7.5)
Mountaineering	36	(4.2)	20	(4.7)	16	(3.7)
Soccer	23	(2.7)	23	(5.4)	0	(0.0)
Kendo	18	(2.1)	15	(3.5)	3	(0.7)
Ski and Skating	14	(1.6)	8	(1.9)	6	(1.4)
Dance	13	(1.5)	0	(0.0)	13	(3.0)
Rugby football	12	(1.4)	12	(2.8)	0	(0.0)
Badminton	7	(0.8)	3	(0.7)	4	(0.9)
Kyudo	6	(0.7)	5	(1.2)	1	(0.2)
Boxing	6	(0.7)	6	(1.4)	0	(0.0)
Ice hockey	4	(0.5)	4	(0.9)	0	(0.0)
Karate	4	(0.5)	4	(0.9)	0	(0.0)
Handball	4	(0.5)	4	(0.9)	0	(0.0)
Rowing	4	(0.5)	3	(0.7)	1	(0.2)
Wrestling	4	(0.5)	4	(0.9)	0	(0.0)
Golf	3	(0.4)	2	(0.5)	1	(0.2)
Equestrian art	2	(0.2)	2	(0.5)	0	(0.0)
American football	1	(0.1)	1	(0.2)	0	(0.0)
Aviation	1	(0.1)	1	(0.2)	0	(0.0)
Weightlifting	1	(0.1)	1	(0.2)	0	(0.0)
Sailing	1	(0.1)	1	(0.2)	0	(0.0)
